# Case Series on Varied Presentations of Amlodipine Toxicity

**DOI:** 10.7759/cureus.83697

**Published:** 2025-05-07

**Authors:** S Sridhar, Sandhiya Narendiran, Murugan K R, A Marimuthu, Sahasyaa Adalarasan, Yogesh S, S Shivamalarvizhi, Hariharan C

**Affiliations:** 1 Internal Medicine, Madras Medical College and Rajiv Gandhi Government General Hospital, Chennai, IND; 2 Internal Medicine, Madras Medical College, Chennai, IND

**Keywords:** amlodipine-, amlodipine toxicity, calcium channel blockers, essential hypertension, hypertension

## Abstract

Calcium channel blockers (CCBs) are among the most widely prescribed drugs for cardiovascular illnesses. Angina pectoris, cardiac arrhythmias, hypertension, and other conditions are treated with calcium channel blockers (CCBs). Both immediate-release and extended-release versions of these drugs are available; the latter are often used in therapeutic settings.

While extended-release CCB overdoses can cause delayed start of dysrhythmias, shock, sudden cardiac collapse, and intestinal ischemia, immediate-release CCB overdoses are characterized by rapid progression to hypotension, bradydysrhythmia, and cardiac arrest. The present case series consists of five cases of amlodipine toxicity that were treated at Rajiv Gandhi General Government Hospital and had a range of clinical manifestations. The present case series also includes two cases of non-cardiogenic pulmonary edema, a rare presentation of amlodipine poisoning.

## Introduction

Calcium channel blockers (CCBs) were first introduced for clinical use in the late 1970s and have since become a cornerstone in managing various cardiovascular and neurological conditions. These drugs act by inhibiting calcium influx through voltage-gated calcium channels, which are essential for muscular contraction and neurotransmission. The calcium channels are classified into L, N, P, T, Q, and R types, with L-type channels playing a particularly significant role in cardiac and vascular smooth muscle physiology [1]. Given their widespread use, understanding both their therapeutic benefits and potential toxicities is essential.

CCBs are broadly categorized into two main classes: dihydropyridines and non-dihydropyridines. Dihydropyridines, including amlodipine, nifedipine, nicardipine, and nimodipine, predominantly act on vascular smooth muscle, making them effective in conditions such as hypertension, Raynaud phenomenon, and Prinzmetal angina [2]. On the other hand, non-dihydropyridines like verapamil and diltiazem exert more pronounced effects on cardiac conduction and contractility, making them valuable in managing atrial fibrillation, supraventricular tachycardia, and myocardial oxygen demand reduction. Despite their therapeutic applications, CCBs, particularly in overdose scenarios, can lead to life-threatening cardiovascular complications.

Amlodipine, a commonly prescribed dihydropyridine CCB, is primarily used to treat hypertension and angina due to its potent vasodilatory effects. However, its excessive intake can precipitate severe cardiovascular toxicity. The primary mechanism underlying CCB toxicity involves excessive inhibition of L-type calcium channels, leading to diminished myocardial contractility, bradycardia, and systemic vasodilation. These effects can culminate in profound hypotension, myocardial depression, and atrioventricular block. Furthermore, since calcium influx is crucial for maintaining vascular tone, its inhibition results in excessive vasodilation and refractory shock, which are hallmarks of severe amlodipine toxicity [3].

Clinically, CCB toxicity manifests with a spectrum of cardiovascular and systemic effects. The most frequent presentation is hypotension, often accompanied by sinus bradycardia, atrioventricular block, and myocardial depression. More severe cases can lead to syncope, altered mental status, and coma, primarily due to CNS hypoperfusion. Metabolic complications, such as hypoinsulinemia-induced hyperglycemia, are also well-documented in CCB overdose. Rare but significant complications include pulmonary toxicity, characterized by interstitial edema and acute pulmonary injury, which may be evident in imaging studies like chest X-rays or ultrasonography [4]. Additionally, patients may exhibit metabolic acidosis, hypoxemia, and evidence of multi-organ dysfunction secondary to systemic hypoperfusion [5].

This toxicity's rare and unusual presentation highlights its distinctive clinical course and management challenges. Previous reports have noted that such atypical cases often pose greater therapeutic difficulties than more common presentations [6]. The complexity arises from the unpredictable progression of symptoms, reduced responsiveness to standard treatment protocols, and potential multi-organ involvement. These factors require a more aggressive and individualized approach to management, often requiring intensive monitoring and advanced support.

## Case presentation

This is a retrospective descriptive case series conducted at Rajiv Gandhi Government General Hospital, reviewing the medical records of patients presenting with amlodipine overdose between 01.01.2024 and 31.12.2024. A total of five patients were included based on the inclusion criteria of confirmed amlodipine ingestion and documented clinical and treatment outcomes.

Case 1

A 60-year-old male, a known hypertensive, presented with an alleged history of ingestion of 50 tablets of amlodipine (5 mg). He presented to us after 4 hours of ingestion. The patient had cold extremities and was alert but lethargic at presentation. His pulse rate was 105 beats per minute, and his blood pressure was 70/40 mmHg. He complained of nausea even after receiving gastric lavage.

After being moved to the intensive care unit, the patient began receiving intravenous fluids. His vital signs were as follows: systolic blood pressure of 70 mmHg, pulse of 126 bpm, respiratory rate of 24 breaths per minute, temperature of 37.5°C, and pulse oximetry showing 98% oxygen saturation under oxygen supplementation. The patient was also mildly lethargic upon physical examination. The head and neck examinations revealed nothing unusual. The cardiac examination revealed no murmurs and normal heart sounds. Auscultation revealed that the lungs were clear. There was no cyanosis or edema, and the extremities were cold. Laboratory test results revealed a few significant findings (Table 1).

**Table 1:** Laboratory findings in Case 1 Hb - Haemoglobin, TLC - Total Leukocyte Count, RFT - Renal Function Test

Investigation	Result	Reference range
Hb	10 g/dL	12-15.5 g/dL
TLC	9300/mm^3^	4000-11000/mm^3^
Urea level (RFT)	32 mg/dL	15-40 mg/dL
Creatinine level (RFT)	1.3 mg/dL	0.5-1.1 mg/dL
HCO_3_^-^	18 mEq/L	23-28 mEq/L
Glucose	93 mg/dL	100-125 mg/dL
Na^+^	138 mEq/L	135-145 mEq/L
K^+^	4.1 mEq/L	3.5-5.1 mEq/L

Although 2000 mL of crystalloids were infused intravenously (after a baseline ECHO, which showed normal systolic function), the mean arterial blood pressure remained below 55 mmHg. The patient was started on inotropic support with noradrenaline. Simultaneously, he was administered a calcium gluconate bolus followed by a maintenance infusion. He also received two doses of glucagon (3 mg subcutaneously over 30 minutes). His systolic blood pressure gradually improved to 90 mmHg. Over the next four hours, there was a sustained increase in systolic blood pressure, and he was gradually weaned off inotropic support. He continued to improve clinically over the next four days and was discharged after a week with stable cardiac function and normal laboratory parameters.

Case 2

A 30-year-old woman was admitted 8 hours after allegedly consuming 80 tablets of amlodipine (5 mg). She had no prior medical history of comorbidities and was previously in good health.

On presentation, her blood pressure was 70/30 mmHg, and her heart rate was 88 beats per minute. She did not exhibit any symptoms of respiratory or cardiac discomfort. Upon admission, the ECG revealed no significant changes and showed a normal sinus rhythm. Her initial chest X-ray was unremarkable or without any significant abnormality, and her laboratory tests were normal. Fluid resuscitation was initiated with continuous monitoring of vitals and urine output, along with simultaneous gastrointestinal decontamination to clear out any remnant amlodipine. After the first hour of resuscitation, due to persistent hypotension, she was started on inotropic support. She was drowsy and lethargic.

The patient worsened gradually over the next 24 hours, becoming tachypneic and dyspneic. Examination at 24 hours revealed bilateral basal inspiratory crepitations. She was managed with a backrest elevation of 30 degrees and oxygen support. Fluid intake was restricted, and a furosemide infusion (1 mg/hr) was initiated along with noradrenaline infusion (0.4 mcg/kg/min), calcium gluconate infusion (1 mEq/kg/hour), and insulin infusion (1 unit/kg/hour). The inferior vena cava (IVC) diameter was monitored daily. CBC, renal function tests (RFT), liver function tests (LFT), and echocardiography remained normal throughout treatment.

She was diagnosed with amlodipine-induced non-cardiogenic pulmonary edema based on the presence of bilateral perihilar infiltrates on imaging, absence of elevated jugular venous pressure, and a normal echocardiogram showing preserved left ventricular function with an ejection fraction of 72%. The lack of clinical or echocardiographic signs of heart failure supported a non-cardiogenic etiology. The patient responded well to diuresis over the following day, her chest findings improved, and she was gradually weaned off oxygen support.

Case 3

A 40-year-old male presented with an alleged history of ingestion of 75 tablets of amlodipine (5 mg) along with 20 tablets of nifedipine (10 mg). He sought medical attention only after 10 hours of ingestion.

On presentation, his vital signs were as follows: heart rate of 112 beats per minute, arterial pressure of 70/40 mmHg, oxygen saturation of 88%, and temperature of 36.4°C. He was drowsy and lethargic. As his Glasgow Coma Scale (GCS) score dropped upon arrival, he was intubated and mechanically ventilated. Bedside ultrasound of the abdomen and echocardiography were within normal limits.

Resuscitation was initiated with intravenous fluids, along with simultaneous gastric lavage. Since he did not respond adequately, he was started on inotropes - initially, noradrenaline, followed by the addition of dopamine. To counteract the competitive blocking of calcium channels, he was administered an infusion of 10% calcium gluconate (4 g/hr) along with insulin infusion (50 units in 45 mL of 25% dextrose for hyperinsulinemic euglycemia), with continuous monitoring of vital signs and laboratory parameters.

After a few hours, the patient's systolic blood pressure began to improve. Following an additional up-titration of insulin to 60 IU/hr (1 IU/kg/hr), with concurrent dextrose administration, the systolic blood pressure increased further. The calcium infusion was tapered and stopped over the next 4 hours. The patient was gradually weaned off inotropic support and insulin infusion. All laboratory parameters remained normal.

After two days, as his GCS improved, he was weaned off mechanical ventilation and extubated. He was discharged on the 14th day with normal renal and cardiac function.

Case 4

A 43-year-old female came with an alleged history of consumption of 50 tablets of amlodipine 5 mg on 3/4/24 at around 4 am at her residence due to a quarrel with her husband. She had four episodes of vomiting and was taken to a nearby hospital. She was admitted and her vitals were stable during her stay. She also had complaints of giddiness and breathlessness - sudden in onset, NYHA grade IV and was referred here on 6/4/24 for further management. On arrival, the pulse rate was 108 beats/min, blood pressure was 110/60 mmHg and oxygen saturation was 89% in room air (later improved to 98% with 4 liters of oxygen). She was conscious, oriented, and afebrile with a GCS of 15/15. A systemic examination was done and the respiratory examination revealed bilateral basal fine crepitation. ECG showed sinus tachycardia.

**Figure 1:**
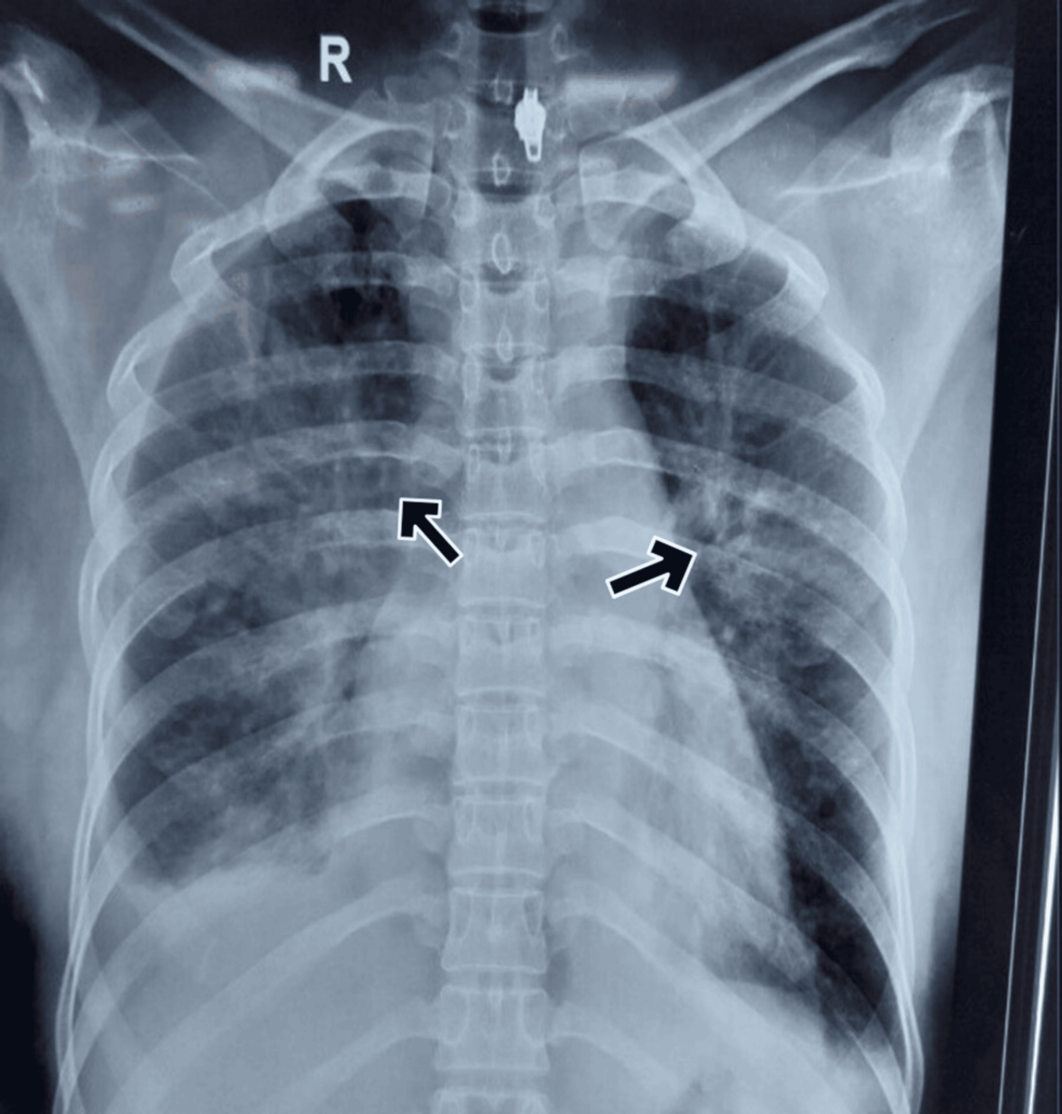
CXR containing bilateral perihilar infiltrates in Case 4

Laboratory investigations revealed a few things about the patient (Table 2). Arterial blood gas analysis indicated respiratory alkalosis. Imaging studies revealed bilateral perihilar infiltrates on chest X-ray (Figure 1), while an abdominal ultrasound demonstrated the presence of bilateral pleural effusion.

**Table 2:** Laboratory findings in Case 4 Hb - Haemoglobin, TLC - Total Leukocyte Count, RFT - Renal Function Test, SGOT - Serum Glutamic Oxaloacetic Transaminase, SGPT - Serum Glutamic Pyruvic Transaminase

Investigation	Result	Reference range
Hb	11.6 g/dL	12-15.5 g/dL
TLC	16400/mm^3^	4000-11000/mm^3^
Platelet count	240k/mm^3^	150k-450k/mm^3^
Urea level (RFT)	32 mg/dL	15-40 mg/dL
Creatinine level (RFT)	0.9 mg/dL	0.5-1.1 mg/dL
Total bilirubin	0.9 mg/dL	0.3-1.2 mg/dL
SGOT	64 IU/L	8-40 IU/L
SGPT	45 IU/L	7-35 IU/L
Na^+^	138 mEq/L	135-145 mEq/L
K^+^	4.1 mEq/L	3.5-5.1 mEq/L

Echocardiography was done which showed normal systolic function with an ejection fraction of 68%. There was progressive tachypnoea and dyspnoea, so the patient was put on continuous positive airway pressure (CPAP) ventilation. CT chest was done for a better understanding and also confirmed bilateral moderate pleural effusion with right more than left, bilateral peribronchial cuffing with perihilar ground glass opacities noted in bilateral lung fields suggestive of pulmonary edema. The patient was treated with furosemide and continued on CPAP ventilation. On 10/4/24, the patient's symptoms improved, and she was weaned from oxygen eventually.

Case 5

A 29-year-old female (with no known comorbidities) presented to the toxicology ICU with an alleged history of consumption of 90 tablets of amlodipine 5 mg. She presented after 4 hours of consumption with complaints of giddiness and lethargy. On examination, the patient was drowsy but arousable. Her vitals at presentation were a pulse rate of 88 beats/min, blood pressure of 80/40 mmHg, and oxygen saturation of 98% in room air. Systemic examination was found to be normal. Gastric decontamination was done immediately after admission. The patient was initially treated with fluid bolus and IVC monitoring, IV calcium gluconate stat doses were given every 10 minutes, followed by calcium gluconate infusion. The patient was started on glucagon infusion and insulin dextrose infusion because of persistent hypotension. She was started on noradrenaline and other supportive measures.

Complete blood count, blood sugar, renal function test, and liver function test were found to be normal. The other laboratory findings aided the treatment plan for the patient (Table 3). The ECG findings were normal. Echocardiography showed normal left ventricular function with an ejection fraction of 72%.

**Table 3:** Laboratory findings in Case 5 RFT - Renal Function Test, ALP - Alkaline Phosphatase, SGOT - Serum Glutamic Oxaloacetic Transaminase, SGPT - Serum Glutamic Pyruvic Transaminase

Investigation	Result	Reference range
Urea level (RFT)	21 mg/dL	15-40 mg/dL
Creatinine level (RFT)	0.8 mg/dL	0.5-1.1 mg/dL
ALP	63 IU/L	30-120 IU/L
SGOT	22 IU/L	8-40 IU/L
SGPT	23 IU/L	7-35 IU/L
Na^+^	136 mEq/L	135-145 mEq/L
K^+^	4.2 mEq/L	3.5-5.1 mEq/L

**Figure 2:**
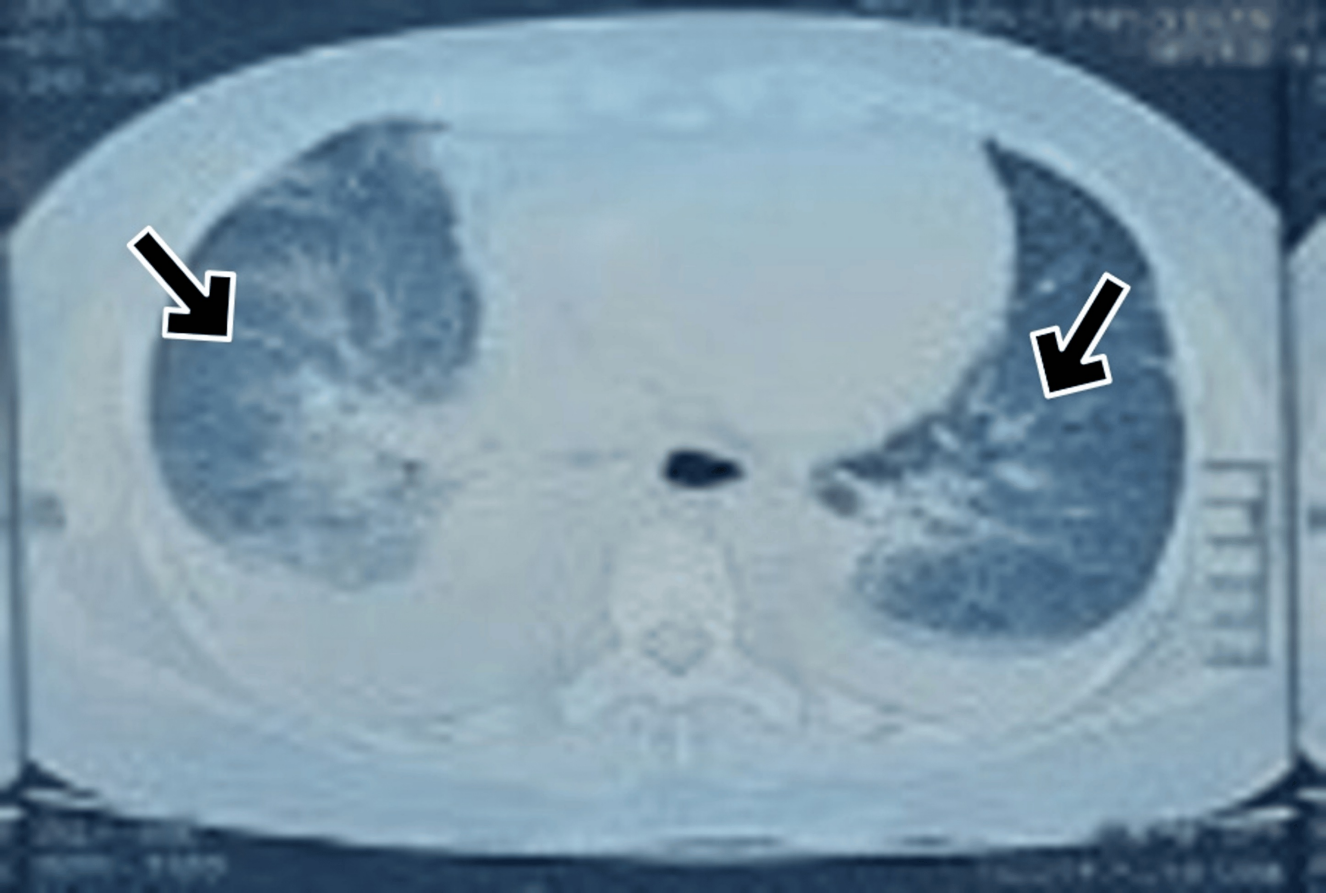
Bilateral pulmonary edema observed in Case 5

The patient gradually worsened over the next 24 hours with complaints of breathlessness. On examination, the patient was tachypneic and dyspneic with SpO2 of 86% in room air. Examination revealed bilateral basal inspiratory fine crepitations. She was diagnosed with acute pulmonary edema. CT chest showed bilateral perihilar infiltrates suggestive of acute pulmonary edema (Figure 2). Ultrasound abdomen showed a normal study with a normal IVC diameter. She was started on oxygen support, furosemide 1 mg/hr, and noradrenaline infusion 0.4 mcg/kg/min. The patient improved after three days with furosemide infusion. The patient was eventually weaned off oxygen support and inotropes over the course of three days.

## Discussion

The public frequently underestimates the potential danger of these substances. One of the most dangerous prescription medication overdoses involves the consumption of large amounts of calcium channel blockers (CCBs). Each of the five scenarios was presented differently, and each case required a distinct management approach. The interval between ingestion and presentation was highly significant, as managing delayed presentations proved challenging.

A clinical diagnosis of calcium channel blocker (CCB) overdose is made based on the patient's history and clinical signs. Treating severe CCB overdose is crucial, as it can be fatal and is often difficult to manage. Previous systematic reviews have provided insight into this [7]. Patients may be in critical condition upon arrival, and those who are not may deteriorate rapidly. In general, supportive care, with continuous monitoring for complications such as bradycardia and hypotension, remains the mainstay of CCB poisoning management. All three patients were discharged after undergoing psychiatric evaluation and counselling. A comparison of all the cases in the present case report is represented below (Table 4).

**Table 4:** Comparison between the different cases

Case	Age / Sex	Dose (mg)	Time Interval	Vitals on Arrival	Interventions	Complications	Outcome
1	60 / M	100	4 hours	BP: 70/40 mmHg, HR: 105 bpm	IV fluids, calcium gluconate, vasopressors	Acute kidney injury	Recovered
2	30 / F	200	8 hours	BP: 70/30 mmHg, HR: 88 bpm	IV fluids, glucagon, calcium chloride	Bradycardia	Recovered
3	40 / M	300	10 hours	BP: 70/40 mmHg, HR: 112 bpm	High-dose insulin euglycemia therapy, calcium gluconate	Hyperglycemia	Recovered
4	43 / F	500	6 hours	BP: 110/60 mmHg, HR: 108 bpm	IV fluids, vasopressors, lipid emulsion therapy	Severe hypotension	Recovered
5	29 / F	700	4 hours	BP: 80/40 mmHg, HR: 88 bpm	Hemodialysis, calcium gluconate, vasopressors	Metabolic acidosis	Recovered

The management of critically ill patients revolves around ensuring airway, breathing, and circulation as primary priorities. In cases of worsening toxicity, endotracheal intubation may be necessary due to the risk of rapid hemodynamic deterioration. If no immediate worsening is observed, patients should be closely monitored in intensive care with continuous cardiac monitoring. The history should emphasize underlying medical conditions, the type of formulation ingested (immediate vs. prolonged release), co-ingestants, and the timing of ingestion. An ECG should be performed to detect conduction anomalies. Initial resuscitation should include intravenous crystalloids, keeping in mind that drug-induced inotropic failure can lead to fluid overload. Therefore, dynamically assessing fluid response through stroke volume or pulse pressure variations may be beneficial. Due to the extensive volume of distribution and lipophilic nature of calcium channel blockers (CCBs), conventional decontamination methods such as hemofiltration, hemodialysis, or urinary alkalinization are ineffective. Whole bowel irrigation remains the primary method of elimination for extended-release formulations. The irrigation could also potentially prevent any intestinal obstruction which was observed in the previous case reports [8].

Gastrointestinal decontamination is debated, particularly in unstable patients, where resuscitation takes priority. The most effective measures for CCB elimination after ingestion include multiple-dose activated charcoal (MDAC) and whole bowel irrigation (WBI) for sustained-release formulations. Activated charcoal should be administered at 1 g/kg over one to two hours, as research has shown that it can reduce amlodipine absorption by 49% if given within two hours. WBI is the preferred decontamination method which is in line with previous literature [9,10]. For substantial sustained-release ingestion, it should be considered for up to four hours. Activated charcoal can also be given at 0.5 g/kg every two to four hours, provided bowel sounds are present and there is no obstruction or perforation.

Calcium administration aims to increase extracellular calcium concentration, promoting calcium influx through unblocked L-type calcium channels. However, responses to calcium therapy are inconsistent and often suboptimal. For adults, an initial intravenous infusion of 13 to 25 mEq of calcium (10-20 mL of 10% calcium chloride or 30-60 mL of 10% calcium gluconate) is recommended, followed by continuous infusion or repeated boluses every 15 to 20 minutes for up to three or four doses. In cases of symptomatic bradycardia, atropine should be administered intravenously at an initial dose of 0.5 to 1 mg (0.02 mg/kg in children, minimum 0.1 mg) every two to three minutes, up to a maximum of 3 mg. In hypotensive patients, epinephrine or norepinephrine is typically the preferred inotropic agent due to its β1-adrenergic activity, which counteracts myocardial depression, while norepinephrine’s α1-adrenergic effects increase peripheral vascular resistance. Pure β-agonists like isoproterenol and dobutamine raise concerns, as their β2-mediated vasodilation can worsen hypotension. Dopamine, which functions as an indirect pressor by inducing norepinephrine release, may be less effective in patients experiencing catecholamine depletion.

Glucagon has inotropic and chronotropic effects and is the preferred treatment for β-blocker overdose, as it bypasses β-adrenergic receptors. However, it offers no significant pharmacologic advantage over conventional β-adrenergic medications in CCB poisoning, as the cellular dysfunction occurs downstream of adenylate cyclase activation. This is also confirmed by previous case reports [11]. The recommended dose for adults is 3-5 mg intravenously over one to two minutes, with an additional 4-10 mg if there is no hemodynamic improvement after five minutes [11]. In children, the initial dose is 50 μg/kg. Hyperinsulinemia euglycemia (HIE) therapy has gained prominence in managing severe CCB toxicity. CCB poisoning shifts myocardial metabolism to rely more on carbohydrates, while also impairing insulin secretion and inducing insulin resistance. Severe poisoning is often associated with significant hyperglycemia. HIE therapy enhances hemodynamic function primarily through increased contractility, with minimal effects on heart rate. Standard treatment begins with an insulin bolus of 1 unit/kg and 0.5 g/kg dextrose, followed by an insulin infusion of 0.5 units/kg/h, titrated up to 2 units/kg/h if no improvement is observed within 30 minutes. Continuous dextrose infusion should be maintained at 0.5 g/kg/h, with glucose levels monitored every 30 minutes for the first four hours. Since insulin’s effects take 15 to 60 minutes to manifest, catecholamine infusions should be initiated beforehand.

In refractory cases, additional hemodynamic support may be required. Case reports suggest that transthoracic or intravenous cardiac pacing can be necessary for increasing heart rate. Intra-aortic balloon counterpulsation has also been considered for cases unresponsive to pharmacologic therapy. However, these interventions are typically available only in tertiary care centers, limiting their widespread application. Overall, the treatment of CCB poisoning requires a multifaceted approach, incorporating early resuscitation, decontamination, targeted pharmacologic therapy, and, in severe cases, advanced circulatory support.

The rare complication of non-cardiogenic pulmonary edema is tackled by diuretics like furosemide. In previous case reports, a similar line of treatment has been followed or prescribed. The rarity can be attributed to the fact that the chances of survival at such high doses could be low due to the hypotensive effect produced by amlodipine. Prevention of further toxicity can be ensured if proper psychiatric counselling is provided.

## Conclusions

Since more and more people are taking calcium channel blockers, amlodipine overdoses are becoming more frequent. Dealing with CCB overdoses requires a methodical approach, and it's critical to understand the potentially fatal consequences. As CCBs have the potential to be fatal, gastric decontamination should be done. Awareness of complications such as hypodynamic shock and acute non-cardiogenic pulmonary edema should be made.

Calcium gluconate infusion to enhance myocardial contractility and systemic perfusion, as well as aggressive fluid resuscitation to maintain blood pressure with precise urine output monitoring, has to be undertaken. If the patient continues to be hypotensive, gets acidotic, or becomes clinically unstable, think about administering insulin infusion and/or inotropic support. When the patient is discharged, make sure they have a psychiatric evaluation and proper follow-up.
